# Enhanced Delivery of Insulin through Acrylamide-Modified Chitosan Containing Smart Carrier System

**DOI:** 10.3390/gels8110701

**Published:** 2022-10-30

**Authors:** Wasmia Mohammed Dahan, Faruq Mohammad, AbdelRahman O. Ezzat, Ayman M. Atta, Hissah Hamad Al-Tilasi, Hamad A. Al-Lohedan

**Affiliations:** Surfactants Research Chair, Department of Chemistry, College of Science, King Saud University, Riyadh 11451, Saudi Arabia

**Keywords:** insulin delivery, modified chitosan, pH-based release, *N*-isopropylacrylamide (NIPAm), 2-acrylamide-2-methylpropane sulfonic acid (AMPS), drug release kinetics

## Abstract

The present study develops on insulin-release studies from the chitosan-amide-modified stimuli-responsive polymers formed from various fatty acids including stearic acid, oleic acid, linoleic acid, and linolenic acid. This is the continuation of an earlier reported study that investigates the insulin-release profiles of chitosan-modified fatty acid amides (without stimuli responsive polymers). Following the synthesis and characterization of many different fatty acid amides with a varying amount of unsaturation, the insulin drug loading and release effects were compared among *N*-isopropylacrylamide (NIPAm), a thermo-responsive polymer, and 2-acrylamide-2-methylpropane sulfonic acid (AMPS), a pH-responsive polymer-modified hydrogel that is expected to enhance environmental response and the controllability of release. Finally, drug release effects were studied to investigate the drug release mechanisms with the help of five different pharmacokinetic models including the zero-order, first-order, Higuchi, Korsmeyers–Peppas, and Hixson models. The results indicate that the Higuchi and Hixson models are valid in terms of the operation of the NIPAm and AMPS matrices during the delivery of insulin.

## 1. Introduction

In recent years, the integration of nanotechnology and pharmacology principles has enabled the development of smart drug delivery systems (DDS) with efficient therapeutics, such as target-organ oriented and patient- and disease-specific drug release [1]. Smart DDS possesses intrinsic control (responding to stimulations) of drug release in specific sites and mitigates the side effects associated with traditional methods without compromise to the release efficacy. These bio responsive systems have proven to be superior in precision medicine, due to several properties such as easy application, local delivery, sustained release, and the ability to improve patient compliance. The polymeric materials provided in the DDS such as hydrogels, dendrimers, liposomes, and micelles, etc., are mostly responsible for such stimuli or environmentally responsive behaviors, such as the temperature, pH, electric shock, magnetic field, ionic strength, etc. [2,3]. Among the various stimuli that have been used, biomolecule-responsive hydrogels, especially those based on natural polymers, have recently attracted attention because of their ability to undergo structural transitions in response to target biomolecules, such as glucose, proteins, nucleic acids, polypeptides, etc. [3]. Hydrogels are crosslinked hydrophilic polymer networks that swell in water as a result of the absorption of fluid, where the content of aqueous solvent within the formulation is increased alongside the size of polymer network, allowing the drug to diffuse through the swollen network to the external environment [4]. As an example, the naturally occurring biopolymer chitosan (CS) is very popular in this category of smart DDS because of the properties of biocompatibility, biodegradability, low toxicity, mucoadhesive feature, and physiological relevance with respect to the medium pH and temperature. However, the biopolymer CS has some limitations in the area of DDS, such as low solubility in neutral and alkaline pH that can be overcome by the surface/chemical modification of functional groups such as primary hydroxyl and amine groups present at the backbone of CS [5,6]. CS, with its bio adhesive nature, can assist in designing drug carrier systems that can bind to the intestinal mucosa and improve the residence time of drugs in the intestinal lumen; consequently, their bioavailability is improved [7]. Additionally, the positively charged surfaces of CS lead to the formation of complex structures with negatively charged drug molecules. Among all the studied stimuli-sensitive materials, polymers that respond to temperature and pH have attracted the most attention because of some important physiological factors in the body and the appearance of a few diseases due to changes in temperature or pH, or even both [6].

Poly(*N*-isopropyl acrylamide) P(NIPAm) is a widely used thermo-responsive polymer and has multiple applications in the biomedical sector because of the interesting property of a lower critical solution temperature (LCST) of 32 °C, which is close to the normal body temperature of 37 °C [8,9]. The heating of P(NIPAm) above LCST leads to polymer breakdown (sharp collapse into a hydrophobic globule), while heating below the LCST leads to the formation of a hydrophilic polymer. This reversible phase transition behavior has been exploited to prepare so-called “smart” drug delivery systems, where the drug encapsulated within a polymeric shell can be released in an intelligent way by slightly increasing the medium temperature [10]. On the other hand, pH-responsive hydrogel polymers are characterized by the presence of acidic or basic ionizable groups, and these groups ionize depending on the pH variation by accepting or releasing protons, thus changing their structure and properties. The pH-responsive polymers maintain the ability to sense even slight changes to the outer environment and respond accordingly by increasing or decreasing the degree of swelling; this ability is particularly useful in oral drug delivery as it can protect the peptide/protein drugs in the gastrointestinal (GI) tract. As an example, poly acidic polymers shrink at a low pH due to the protonation of acidic groups and become unionized so as to protect the drug along the stomach; at the same time, it swells/expands at a high pH, which leads to the release of the loaded drug [11]. Polymers containing a sulfonic acid group are usually preferred in the preparation of hydrogels where the sulfonate group provides the monomer with a high degree of hydrophilicity in an anionic form; the most widely used polymers of this category are poly(2-acrylamido-2-methylpropane sulfonic acid) (PAMPS) and poly(4-styrene sulfonic acid) [12].

Diabetes mellitus (DM) is a disorder caused by the decreased production of insulin or the decreased ability to use the produced insulin, which is a polypeptide hormone produced by the pancreatic cells. The hormone potentially coordinates with glucagon to modulate blood glucose levels where insulin acts through the anabolic pathway while glucagon performs catabolic functions [13]. Insulin is a common medicine used to treat both Type 1 and advanced Type 2 diabetes; the common route of administration is through injection; however, this is a painful method of administration and may cause problems with compliance to treatment. Administration via the oral route can overcome poor compliance of the drug; however, this drug has low stability in the GI tract and its hydrophilic nature supports enzyme degradation. Furthermore, it is poorly absorbed in the intestines due to its large size [14]. Therefore, in order to protect insulin in an acidic medium and prevent it from being degraded via digestion by enzymes of the stomach and intestines, carrier materials such as CS can be used. The usage of CS in its pure and/or cross-linked form has the tendency of leading to enhanced loading and stable coating whereby loaded drugs are released in a controlled manner and absorbed effectively into the bloodstream through the intestines [15].

In this view, the encapsulation of insulin within the biocompatible matrices of a cross-linked CS nano carrier formed by the treatment of polycationic CS with that of negatively charged tripolyphosphate (TPP) has been found to effectively protect the drug from GI pH, enzymes, and at the same time enhance bioavailability and absorption due to the nano size of DDS. Taking this point into consideration, another DDS-containing CS-fatty acid amide grafted with thermo-response polymers and cross linked with TPP has been found to be highly efficient in the oral delivery of insulin [16]. However, the limitation of these studies is that the release was only tested for a short period of 4 h at two different pHs of 1.2 and 7.4. To verify these findings, it can be useful to investigate the release profiles with a sustainable carrier system over longer periods (up to 24 h) so as to analyze the adjustments in the blood and body glucose levels.

Keeping the structural advantages of CS and its crosslinked form as a sophisticated nanocarrier in mind, our aim is to develop a DDS with controlled properties that can enhance the oral bioavailability of insulin over extended time periods. In this view, one of our earlier studies investigated insulin release from CS and its cross linked amide forms, where the nano/micro sized CS hydrogels were formed by the ionic gelation route, which is simple, convenient and does not require reactive/toxic solvents and high-temperature heating conditions. For the study, the chemical structure of CS was modified with four different fatty acids of saturated and unsaturated origin since the fatty acids serve as important components of body cells, where the amide coating on CS is expected to form a hydrophobic layer that prevents both the drug and carrier material from degradation [17,18]. In addition, it has been hypothesized that the drug-carrying polymer creates a disorder and thus leads to increased permeability when inserted into the phospholipid bilayer of the cell membrane [19]. To develop on our earlier study, the present work investigates insulin release behaviors from the fatty acid amides of CS additionally conjugated with two different stimuli responsive polymers, 2-acrylamide-2-methylpropane sulfonic acid (AMPS) for pH sensitivity and *N*-isopropylacrylamide (NIPAm) for temperature sensitivity. Thus, the formed CS grafts were subjected to cross linking in the presence of TPP to enrich the pH and temperature sensitivities, enhance the drug absorption rate, and to control/stabilize insulin release. The fabricated hydrogel matrices were characterized by making use of various techniques that includes the TEM, FTIR, TGA, UV-Vis, and DLS analyses. Following characterization, insulin release studies were performed to observe the changes of pH in the solution. We found the pH to be similar to that of the GI tract, where UV-Vis spectroscopy at 284 nm was used for the qualitative and quantitative analysis of insulin loading and release. Finally, the insulin release kinetics were evaluated against five different pharmaco-kinetic models (zero order, first-order, Higuchi, Korsmeyers–Peppas, and Hixson models).

## 2. Results and Discussion

### 2.1. Physicochemical Characterization

In this work, we studied and compared the insulin release behaviors from carrier material made up two different stimuli-responsive-polymer (NIPAm and AMPS) modified CS-fatty acid amides (CS-g-OA, CS-g-LA, CS-g-LLA, and CS-g-SA) with cross linking. For the study, the NIPAm- or AMPS-modified CS-amides (from fatty acids of SA, OA, LA, and LLA) were first synthesized, followed by physicochemical characterization to determine their functionality, insulin drug loading, thermal stability, surface charges, particles size, and morphological changes.

**Figure 1:**
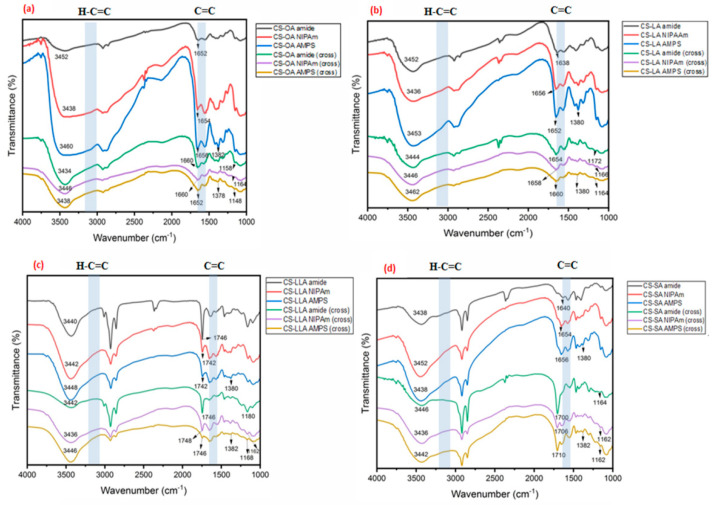
FTIR analysis of CS-amide, CS-amide-modified NIPAm or AMPS matrices before and after crosslinking; (**a**) OA; (**b**) LA; (**c**) LLA; and (**d**) SA.

Figure 1 shows the comparison of FTIR spectrums of various CS-based fatty acid amides before and after modification with NIPAm or AMPS and with or without crosslinking with TPP. In this, the FTIR analysis specifically identifies the presence of amide groups and phosphate groups as a means of confirmation for the occurrence of amidation and cross-linking reactions. The disappearance of double bonds (C = C) and (H-C = C) from the acryl groups has also been observed and the broad band peaks at 3350–3500 cm^−1^ referred to -OH and -NH stretching frequencies. The bands observed at around 1638 to 1748 cm^−1^ referred to the -C = O stretch in the –CONH in all samples, while the bands in the range of 1378–1382 referred to S = O in the CS-AMPS samples. The disappearance of peaks at 3100 cm^−1^ of H-C = C and 1620 cm^−1^ in the C = C stretch of acryl monomers in the CS-AMPS/NIPAm hydrogel spectrum indicated the Michael addition of acryl monomers to the amino groups of CS. In addition, the cross-linked samples showed the same functional groups as non-cross-linked ones with small noticeable band shifts, alongside the appearance of new bands in the range of 1148 to 1180 cm^−1^ relating to the P = O bonds of TPP cross-linker [20].

**Figure 2:**
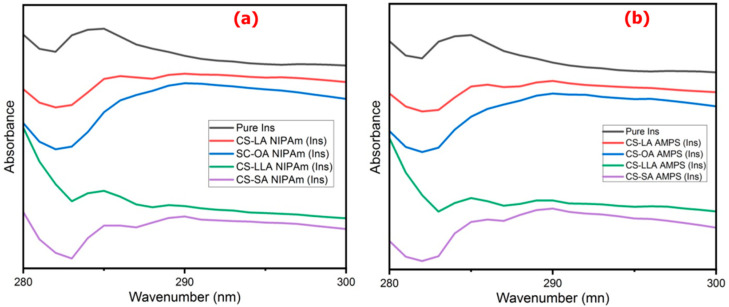
Comparison of UV-Vis spectrums for pure insulin and insulin-loaded CS-amides-modified (**a**) NIPAm; and (**b**) AMPS.

UV-Vis spectroscopy was used to confirm the loading of insulin onto the as-prepared hydrogel matrices, for which the respective analysis is shown in Figure 2a,b. Insulin has an absorption maxima λ_max_ of 284 nm and the same was determined by scanning the drug-containing solution with UV-Vis spectrophotometer over a range of wavelengths (200–500 nm), where distilled water was used as blank. As can be seen from the analysis, both pure insulin and insulin-loaded hydrogel matrices demonstrated the maximum absorption at the wavelength range of 280 to 290 nm, thereby indicating the successful loading of insulin within the hydrogel matrices. In the graph, which shows a comparison of the pure insulin spectrum with those of the other four samples, the absorption maxima can be observed for all; however, a change of intensity was observed due to the different levels of drug loading on each hydrogel matrix.

**Figure 3:**
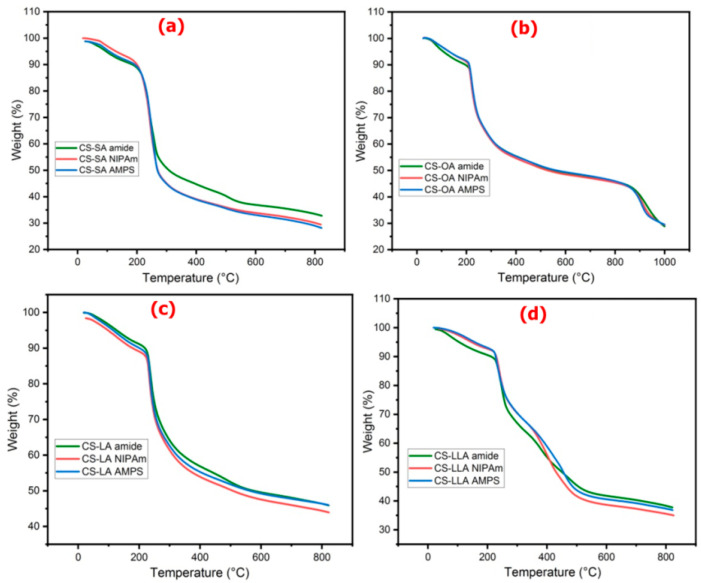
TGA comparison of CS-amides-modified NIPAm and AMPS; (**a**) SA; (**b**) OA; (**c**) LA; and (**d**) LLA.

The TGA of CS-amides compared to that of NIPAm and AMPS-modified CS amides are shown in Figure 3, where the dissociation studies with temperature changes were carried out in the heating range of 25 to 800 °C. By carefully observing TGA graph, it can be seen that all the prepared samples decomposed in two main steps; the first step involves temperatures of up to 150 °C and indicates the loss of residual or physically adsorbed water in the polymer network [21]. The percentage of water trapped in the polymer networks was slightly higher in the grafted samples with AMPS and NIPAM, as these monomers increased the hydrophilicity of as-prepared polymers with cross-linking. In the same way, the second decomposition step that took place in the temperature range of 150 to 600 °C was found to be slightly higher in terms of the residual percentage of polymers after grafting with AMPS and NIPAM. This is because both AMPS and NIPAM have some polar groups (including amide and sulfonyl in the case of AMPS, while only amide group in NIPAM) that are able to form hydrogen bonds and electrostatic interactions in the structure, which lead to more effective cross-linking and increase the stability of polymer chains [22]. However, they lower the intra- and inter-molecular hydrogen bond of CS and as a result, a decrease in thermal stability is observed.

**Figure 4:**
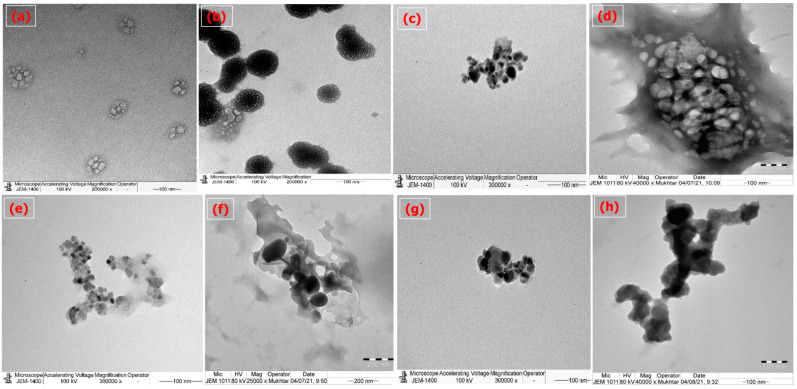
TEM analysis of (**a**) CS-g-SA-modified AMPS; (**b**) CS-g-OA-modified AMPS; (**c**) CS-g-LA-modified AMPS; (**d**) CS-g-LLA-modified AMPS; (**e**) CS-g-SA-modified NIPAm; (**f**) CS-g-OA-modified NIPAm; (**g**) CS-g-LA-modified NIPAm; and (**h**) CS-g-LLA-modified NIPAm.

The surface morphologies of different CS-based NPs, as-prepared by the ionic interaction between different CS derivatives and negatively charged phosphate groups of TPP, were investigated by performing a TEM analysis and are shown in Figure 4a–h. The TEM micrographs revealed that the modified CS NPs appeared as solid spheres; from the particle size analysis, it was found that the AMPS-modified CS NPs (Figure 4a–d) possess are of a smaller size than those modified with NIPAM (Figure 4e–h). The latter demonstrated the formation of some agglomerations. The possible adsorption and binding of AMPS monomers to the CS surface via hydrogen bonding between the protonated amino groups of CS and sulphonic acid groups of AMPS may explain these results [23].

**Figure 5:**
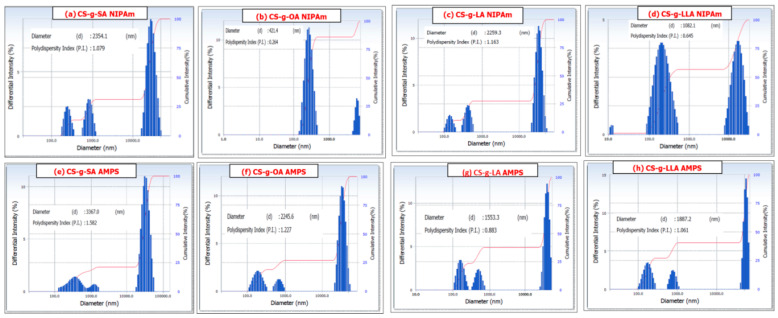
DLS measurements for CS-modified amide with NIPAm and AMPS nanoparticles.

The DLS analyses were used to measure the particle diameter (in solution phase), PDI, and zeta potential of different CS derivatives in order to determine the size distribution in solution, and the surface charge of the prepared nanogels (Figure 5 and Table 1). We found that most of the particles fell in the size range of several micrometers due to the swelling of gels when they dropped into the solution. The analysis indicates that most of the prepared CS derivatives have positive surface charges due to the cationic nature of protonated primary amino groups on the CS molecule. Having a charged surface improves the insulin-loading capacity as a result of ionic bonding with the insulin molecule as its structure has -COO^−^ and NH^+^ groups. The positive charges provide protection for the insulin drug against stomach fluid and enhance the uptake of intestinal cells; therefore, the delivery can be easily controlled by the target sites [18]. Furthermore, the cross-linked networks act as a bowl entrapping the drug and control its release [24].

### 2.2. Studies of Insulin Drug Loading and Release

**Figure 6:**
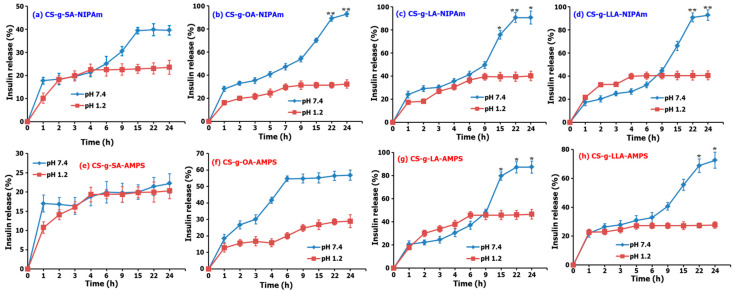
Studies of insulin release (%) at two different pHs of 1.2 and 7.4 for the CS-based fatty acids (SA, OA, LA, and LLA) modified with NIPAm (**a**–**d**) and AMPS (**e**–**h**). * corresponds to the significant and ** for the highly significant values at probability *p* < 0.05 and *p* < 0.01, respectively.

Figure 6 and Table 2 provide the comparison of insulin-loading capacity (%) as a measure of encapsulation efficiency (EE%) along with equilibrium time (ET) and release (%) for the NIPAm- or AMPS-modified CS-fatty acid amide matrices. Comparing CS-amides by different fatty acids with those ones modified with NIPAm and AMPS, in a general sense, revealed an improvement in the loading capacity for most samples, i.e., NIPAm and AMPS functional groups enhance the loading capacity of cross-linked hydrogels. Among the tested samples, CS-g-LLA-NIPAm and CS-g-OA-AMPS have the highest and similar EEs of 85.5%, followed by CS-g-LLA-AMPS, CS-g-SA-NIPAm, and so on. The lowest EE is exhibited by the CS-g-OA-NIPAm sample, with 60.8%, and is comparatively higher than that of CS-based samples without NIPAm or AMPS modification, which is CS-g-LA 55.95% [17], thereby providing evidence for enhanced insulin-loading with NIPAm or AMPS modification.

During further comparisons of insulin release (%) at two different pHs (1.2 and 7.4), both the NIPAm and AMPS-modified CS-fatty acid amide samples showed an enhanced release capacity at the neutral pH of 7.4 as compared to the acidic pH of 1.2. In the release studies, the NIPAm-modified CS samples proved to be better releasing agents (than AMPS-modified ones) with the highest releases of 92.9% (for CS-g-OA-NIPAm with EE of 60.8% and ET of 24 h) and 92.8% (for CS-g-LLA-NIPAm with EE of 85.5% and ET of 24 h) at the neutral pH of 7.4. However, for the AMPS-modified samples at a pH of 7.4, the highest release was noted for CS-g-LA-AMPS with 87.4% (from the EE of 76.6% and ET of 15 h) and lowest release of 22.2% for CS-g-SA-AMPS (78.6% EE and ET of 6 h). For the CS-g-OA-AMPS sample, although observed to have an EE of 85.5%, the release at pH 7.4 was noted to only have an EE of 56.8% and ET of only 7 h. Therefore, from the comparison of all the insulin-loading and release data onto the matrices of NIPAm or AMPs-modified CS, the CS-g-LLA-NIPAm sample was confirmed to be the superior drug-releasing agent with its highest EE and ET at pH 7.4. Additionally, by comparing these results with earlier published data on CS-modified fatty acid samples (CS-g-SA, CS-g-OA, CS-g-LA, and CS-g-LLA) without stimuli polymer (NIPAm and AMPS), it is clear that the addition of polymers significantly enhanced the drug-loading capacity of all hydrogel matrices [17]. For example, the EE observed for the pure CS-g-LLA hydrogel sample was 83.3%, while for CS-g-LLA-NIPAm it is 85.6%; which this method of insulin loading, the release rates of CS-g-LA and CS-g-LLA-NIPAm are 86.7% and 92.8%, respectively (pH 7.4). Similarly, for CS-g-LA-AMPS at a 76.6% insulin loading, a release rate of 87.4% at pH 7.4 was observed.

In general, for pharmakokinetic studies that deal with ET, most samples release about 20% during the first 1 h; in the present study, the CS-g-SA-AMPS and CS-g-OA-AMPS samples reached their ETs quite rapidly, i.e., within the first 6 and 7 h, respectively. This observation indicates that the CS-g-SA-AMPS and CS-g-OA-AMPS carriers may serve as promising releasing agents for drugs that need to be delivered quickly, i.e., as soon as they are administered into the body, such drugs used in the treatment of heart attack and lung disorders. In a similar way, the CS-g-SA-NIPAm and CS-g-LA-AMPS samples displayed a moderate ET of 15 h, and the rest of the samples demonstrated a prolonged ET of 20–24 h. Prolonged ETs are particularly useful to achieve our present goal of keeping the rate of glucose in the blood low in diabetic patients, with the continuous release of insulin for the 24 h period after the administration.

### 2.3. Insulin Release Kinetics and Mechanism of Action

**Figure 7:**
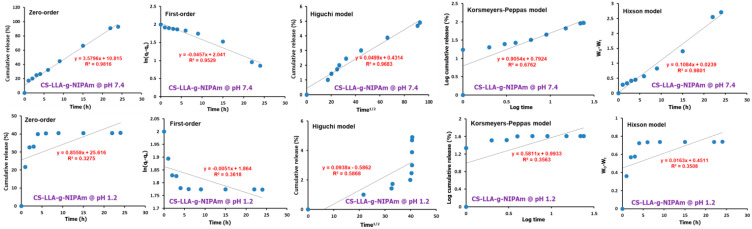
Kinetic study parameters of CS-LLA-g-NIPAm at two different pHs of 1.2 and 7.4.

**Figure 8:**
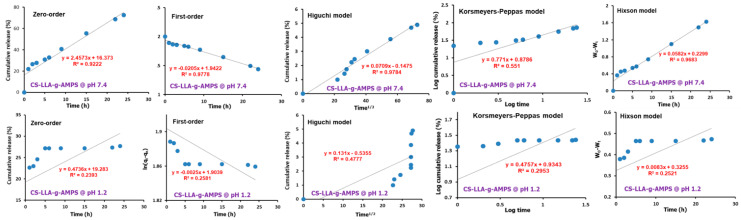
Kinetic studies of CS-LLA-g-AMPS at pH 1.2 and 7.4.

To understand the insulin-release mechanism for CS-based fatty acid amides modified with NIPAm or AMPS matrices (with cross-linking), the drug delivery data were tested against five different pharmacokinetic studies including zero-order, first-order, Higuchi, Korsmeyers–Peppas, and Hixson models (Table 3). When comparing the obtained data, it might be confusing to discuss all the information, i.e., eight samples with five different kinetic models, and at two pHs; therefore, we focused mainly on the NIPAm- or AMPS-modified CS-g-LLA samples (Figure 7 and Figure 8, while other graphs are presented in the Supporting Information (Figures S1–S6)). Based on the correlation coefficient (R^2^) values provided in Table 3, the insulin release from the CS-g-LLA-NIPAm sample generally follows the zero-order (0.981), Higuchi (0.968) and Hixson models (0.980) at pH 7.4. This means that the insulin release from the CS-g-LLA-NIPAm matrix delivers a constant amount of the drug per unit time (as per zero order), in addition to release through diffusion (based on Higuchi) and dissolution (Hixson model). However, for the CS-g-LLA-AMPS sample, the R^2^ values at pH 7.4 indicate that the insulin release follows both the first-order (0.977), Higuchi (0.978), and Hixson models (0.968). The release by first-order kinetics provides information that the release rate is strongly dependent on drug concentration (as per first-order) and also occurs through diffusion (Higuchi) and dissolution processes (Hixson). In addition, in comparison with the insulin-release kinetics of the pure CS-g-LLA matrix, where it follows only the Higuchi kinetic model [17], the drug release of the CS-LLA-g-NIPAm matrix occurs through three independent mechanisms (zero-order, Higuchi, and Hixson). This provides information that for the successful release of insulin with the usage of CS-g-LLA-NIPAm matrix, any of the mechanisms can be sufficient for the efficient release, as compared to pure CS-g-LLA with only one single pathway. During further comparison of the drug-release mechanisms among the polymers of NIPAm and AMPS, we found that the NIPAm modification to CS-LLA leads to release through the dissolution mechanism, while the AMPS modification to the same CS-LLA carrier leads to the diffusion-mediated process.

The present investigation was carried out to identify an effective carrier system for the controlled delivery of insulin. To achieve this, CS-amides were formed from four different fatty acids (SA, OA, LA, and LLA) with varying amounts of unsaturation. From the insulin-release studies of these CS-fatty acid amides with TPP cross-linking, the CS-g-LLA was found to be superior among all other samples when tested at a solution pH of 7.4. Furthermore, the analysis was carried out to test the influence of insulin release following the incorporation of two different stimuli-responsive polymers, NIPAm and AMPS.

The drug-release profiles of thermo-responsive CS-amide-modified NIPAm nanogels were investigated at 37 °C as multiple factors protect the drug under harsh conditions and improve release. In this view, the outer layer of fatty acids and NIPAm acts as a protective shield for the drug which is electrostatically trapped in the matrix. This also reduces the positive charge on the surface of the carrier matrix by becoming hydrophobic with long chains of acyl groups [18]. When the intestinal cells absorb the capsule, the cellular fluid enters the nanogel mesh, facilitates swelling, and releases the insulin drug through the occurrence of diffusion from the high-concentration side (capsule) to the lower side (cell). The lower critical solution temperature (LCST) of p(NIPAm) is in the range of 32 °C; above this temperature (in our case 37 °C), the capsule begins to collapse and the insulin drug is released from the matrix network with time. In addition, the dietary enzymes also play a critical role in releasing the drug by causing the outer shell to degrade without the need to remove the materials after treatment.

**Scheme 1:**
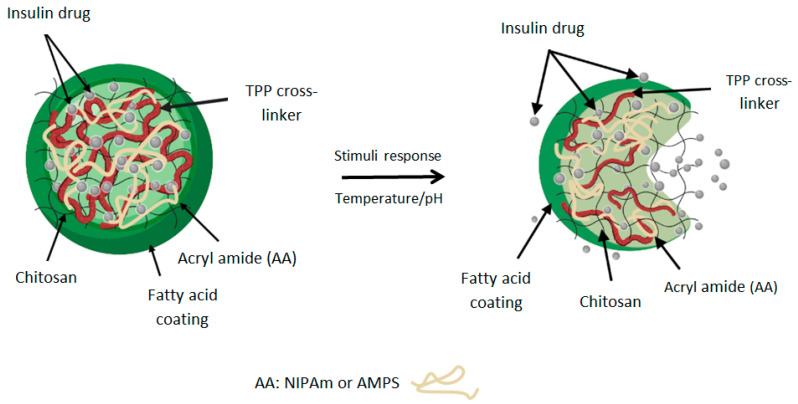
Schematic representation of the insulin release mechanism from the CS-amide-stimuli responsive polymeric system.

In the case of CS-amide-modified AMPS nanogels, the sulfonic acid groups are able to sense changes in the pH environment and react. At a low pH of 1.2 that allows for easy protonation, the surface becomes positively charged and protects the drug from intestinal fluids. The positively charged CS shell surface consists of long-chain acyl groups that encapsulate the drug and slows down its release. However, the observation of better insulin release rates at pH 7.4 may be due to the enhanced interaction between the alkaline solvent and the positively charged CS shell where the solvent infiltrates further inside the nanogel through diffusion. The swelling of nanogels to an ionic state at an alkaline pH also leads to greater insulin release. The poly-acidic polymer shrinks at a low pH as the acidic groups become protonated and unionized to protect the drug through the stomach and swell/expand at a high pH, leading to drug release [24]. An illustration of the insulin-release mechanisms from the CS-based fatty acid amide matrices with NIPAm or AMPs modification is provided in Scheme 1.

## 3. Conclusions

In conclusion, the present study investigated insulin release effects in CS-fatty acid amide matrices modified with two different stimuli-responsive polymers, NIPAm and AMPS. From the comparison of insulin-release studies, NIPAm, with its thermo-responsive nature, was found to be a superior operating mechanism for controlled and sustained insulin delivery from the CS-LLA matrix with TPP cross-linking, in contrast to the AMPS polymer which has a pH-responsive nature. Thus, considering this point of view, the developed CS-LLA-g-NIPAm matrix is ideal for insulin delivery in patients suffering from diabetes, where the normal body temperature of 37 °C is sufficient to generate thermal stimuli that supports controlled insulin delivery for prolonged time periods (viz 20–24 h).

## 4. Experimental

### 4.1. Materials and Methods

The high molecular weight chitosan (CS), the four fatty acids—i.e., oleic acid (OA; 65–88%), linoleic acid (LA; ≥99%), linolenic acid (LLA; ≥99%), and stearic acid (SA; 95%)— *N*-Isopropylacrylamide (NIPAm; 99%), Acrylamido-2-methyl-1-propanesulfonicacid (AMPS; 99%), methanol (99.8%), and dichloromethane (99.9%) were purchased from Sigma-Aldrich (Jinan, China). The other chemicals, including acetic acid (99%), 1-ethyl-3-(3-dimethylaminopropyl) carbodiimide (EDC), which was used as catalyst, and sodium tripolyphosphate (TPP), used as the cross linker, were obtained from Loba Chemie (Mumbai, India). Insulin from NOvoRapid^®^ FlexPen (Bagsværd, Denmark), with each mL containing 100 units of insulin, was used for loading. A dialysis membrane (MWCO 3500) was obtained from Thermo Scientific (Rockford, IL, USA) and the deionized water was used across all treatments. All chemical were used without further purification.

### 4.2. Synthesis of Smart Hydrogels

Step 1: Synthesis of CS-fatty acid amides

The first step involves the formation of CS amides by the individual reaction of four different fatty acids with CS in the presence of EDC. The complete description of the synthesis procedure is provided in our earlier work [17].

Step 2: Modification of CS amides with acrylamides

For the modification, 1 g of CS-g-fatty acid amides was dissolved individually in 50 mL of distilled water containing 0.5 mL of acetic acid. To this solution, 2 g of either of the acrylic reagents (NIPAm or AMPS) was added and subjected to stirring at 50 °C for 2 days; afterwards, ammonium hydroxide was added to precipitate the corresponding hydrogel.

Step 3: Cross-linking of CS-amide-modified acrylamides

**Scheme 2:**
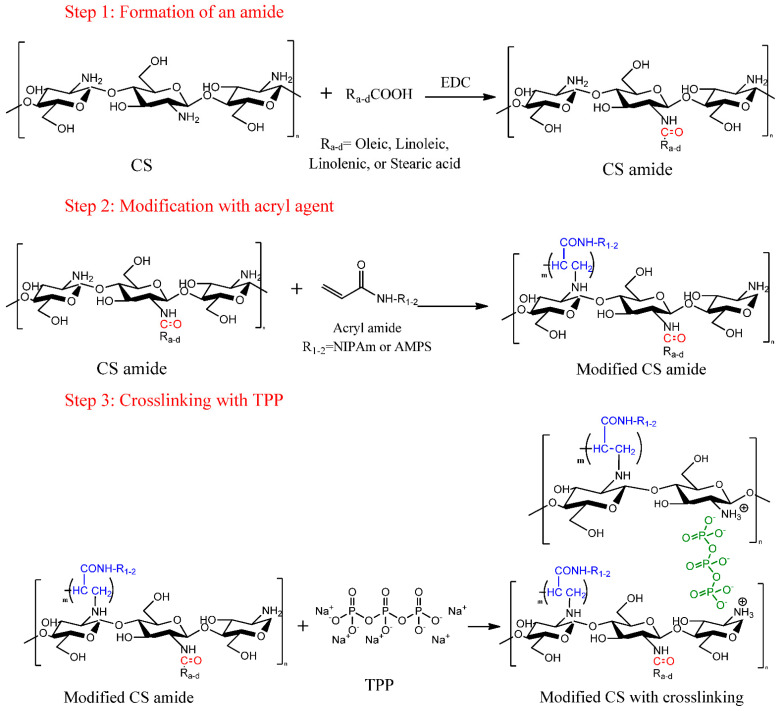
Synthesis steps for the formation of cross linked CS-modified acrylamide hydrogels from pure CS.

To perform cross linking, 0.25 g of CS-amide-modified acrylamide hydrogel polymer was first dissolved in 50 mL of 0.1 M acetic acid, followed by the addition of methylene chloride (5%, *v/v*) and then subjected to homogenization for 2 min at 12,000 rpm. Afterwards, methylene chloride was separated using a rotary evaporator under vacuum at 25 °C and at a 450 atm pressure for 30 min. Then, 10 mL of TPP (8% *w/v*) solution was added under constant stirring at room temperature where mixing was continued for an additional 30 min. Following this, the precipitated hydrogel was separated out by performing filtration, washed with distilled water 2–3 times, and dried in vacuum for 48 h to obtain the dried powder. Scheme 2 illustrates the 3 steps involved in the formation of CS-based acrylamide matrices.

### 4.3. Instrumental Analysis

A Fourier transform infrared spectroscopy (FTIR) analysis of acrylamide-modified CS samples at different stages of their formation was performed with a Nexus 6700 FTIR instrument (Nicolet Magna Newport, NJ, USA). For the UV-Visible spectroscopic analysis, a Perkin Elmer Lambda 45 spectrometer (American Laboratory Trading, San Diego, CT, USA) at an absorption wavelength of 284 nm (insulin drug) was used. In this step, several standard solutions containing insulin were first prepared with known concentrations and at a constant volume (3 mL). The absorbance (λ_max_ of 284 nm) was then measured to draw the calibration plot and, from there, the insulin loadings on the hydrogels were obtained. Additionally, dynamic light scattering (DLS) studies were performed to measure the particles size, polydispersity index (PDI), and zeta potentials of all the prepared samples, for which a Nanoplus Zeta Potential and Nano Particle Analyzer (Malvern Instruments, Malvern, UK) was used. For the thermogravimetric analysis (TGA), a PerkinElmer TGA-7 Thermo gravimetric Analyzer (American Laboratory Trading, San Diego, CT, USA) in the temperature range of 25 to 800 °C was used. A transmission electron microscopy (TEM) analysis was performed for the morphological and particles distribution and, for that, a JEM 1400 Plus–HC FC instrument with standard sampling procedures was used. The insulin drug-loading and release studies were performed as per the procedure reported previously [17], where the encapsulation efficiency (EE), equilibrium time (ET), and percentage of drug release were analyzed at two different pHs of 1.2 and 7.4; these values were chosen based on the physiology. All drug-loading and release experiments were repeated thrice and the data shown are the mean ± standard deviation (SD) of three individual studies, where the statistical analyses were performed with one-way ANOVA (analysis of variance).

## Figures and Tables

**Table 1:** Comparison of the data of particle size, PDI, and zeta potential for various CS-based modified samples.

Sample	Particle Size (nm)	PDI	Zeta Potential (mV)
CS-g-SA-NIPAm	2354	1.07	−14.52
CS-g-OA-NIPAm	421	0.26	10.10
CS-g-LA-NIPAm	2259	1.16	6.10
CS-g-LLA-NIPAm	1082	0.64	−15.51
CS-g-SA-AMPS	3367	1.5	22.75
CS-g-OA-AMPS	2245	1.2	−7.50
CS-g-LA-AMPS	1553	0.88	11.57
CS-g-LLA-AMPS	1887	1.06	11.12

**Table 2:** Comparison of % encapsulation efficiency (EE), equilibrium time ET (h), and release study of CS-based NIPAm and AMPS matrices at two different pHs for the insulin drug.

Sample	EE (%)	ET (h)	Insulin Release (%)	
			pH 7.4	pH 1.2
CS-g-SA-NIPAm	84.7 ± 2.1	15	39.5 ± 2.1	23.5 ± 3.3
CS-g-OA-NIPAm	60.8 ± 2.7	24	92.9 ± 2.2	32.4 ± 3.5
CS-g-LA-NIPAm	66.6 ± 3.5	20	90.7 ± 5.5	40.1 ± 4.2
CS-g-LLA-NIPAm	85.6 ± 4.1	24	92.8 ± 4.5	40.6 ± 3.9
CS-g-SA-AMPS	78.6 ± 2.8	6	22.2 ± 2.5	20.3 ± 2.1
CS-g-OA-AMPS	85.5 ± 3.1	7	56.8 ± 3.1	28.9 ± 3.9
CS-g-LA-AMPS	76.6 ± 3.3	15	87.4 ± 5.2	46.6 ± 4.1
CS-g-LLA-AMPS	84.9 ± 2.4	24	72.6 ± 5.5	27.6 ± 2.2

**Table 3:** Correlation coefficient (R^2^) comparison of insulin release from various CS-fatty acid amide derivatives with various pharmacokinetic models at two different pHs.

Sample	Zero-Order		First-Order		Higuchi Model		Korsmeyers–Peppas Model		Hixson Model	
	pH 7.4	pH 1.2	pH 7.4	pH 1.2	pH 7.4	pH 1.2	pH 7.4	pH 1.2	pH 7.4	pH 1.2
CS-SA-g-NIPAm	0.77	0.349	0.827	0.367	0.935	0.605	0.541	1	0.81	0.362
CS-OA-g-NIPAm	0.919	0.523	0.961	0.566	0.984	0.779	0.537	0.443	0.976	0.553
CS-LA-g-NIPAm	0.938	0.541	0.979	0.588	0.984	0.786	0.58	0.472	0.984	0.574
CS-LLA-g-NIPAm	0.981	0.327	0.952	0.361	0.968	0.586	0.676	0.356	0.98	0.35
CS-SA-g-AMPS	0.363	0.376	0.388	0.394	0.602	0.637	0.368	0.405	0.381	0.389
CS-OA-g-AMPS	0.572	0.707	0.629	0.755	0.802	0.902	0.505	0.499	0.612	0.741
CS-LA-g-AMPS	0.939	0.458	0.978	0.511	0.971	0.717	0.64	0.434	0.974	0.494
CS-LLA-g-AMPS	0.922	0.239	0.977	0.258	0.978	0.477	0.551	0.295	0.968	0.252

## Data Availability

The data are available upon request from the first/corresponding author.

## Supplementary Materials

The following supporting information can be downloaded at: https://www.mdpi.com/article/10.3390/gels8110701/s1, Figure S1: Kinetic studies of CS-SA-g-AMPS. Figure S2: Kinetic studies of CS-SA-g-NIPAm. Figure S3: Kinetic studies of CS-OA-g-AMPS. Figure S4: Kinetic studies of CS-OA-g-NIPAm. Figure S5: Kinetic studies of CS-LA-g-AMPS. Figure S6: Kinetic studies of CS-LA-g-NIPAm.
