# Supplementary material for: Enhanced Delivery of Insulin through Acrylamide-Modified Chitosan Containing Smart Carrier System
Source: Gels. 2022 Oct 30;8(11):701. doi: 10.3390/gels8110701 (PMC9689140; doi:10.3390/gels8110701)
Supplement: Supplementary file 1 [file gels-08-00701-s001.zip › gels-1927583-supplementary.pdf]

Supporting Information

Enhanced Delivery of Insulin through Acrylamide-Modified Chitosan Containing Smart Carrier System

Wasmia Mohammed Dahan, Faruq Mohammad, AbdelRahman O. Ezzat, Ayman M. Atta, Hissah Hamad Al-Tilasi and Hamad A. Al-Lohedan *

Surfactants Research Chair, Department of Chemistry, College of Science, King Saud University, PO Box 2455, Riyadh 11451 Kingdom of Saudi Arabia

* Correspondence: hlohedan@ksu.edu.sa; Tel: +955-114675991

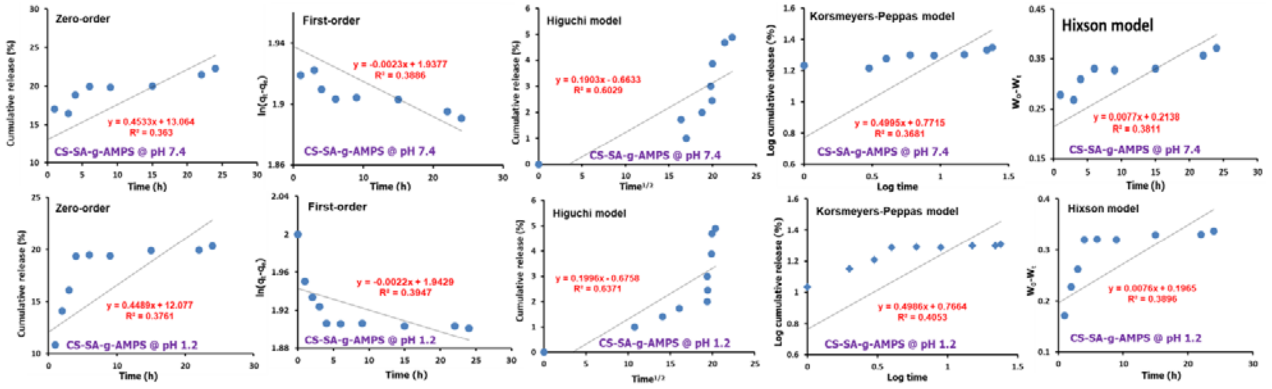

Figure S1: Kinetic studies of CS-SA-g-AMPS.

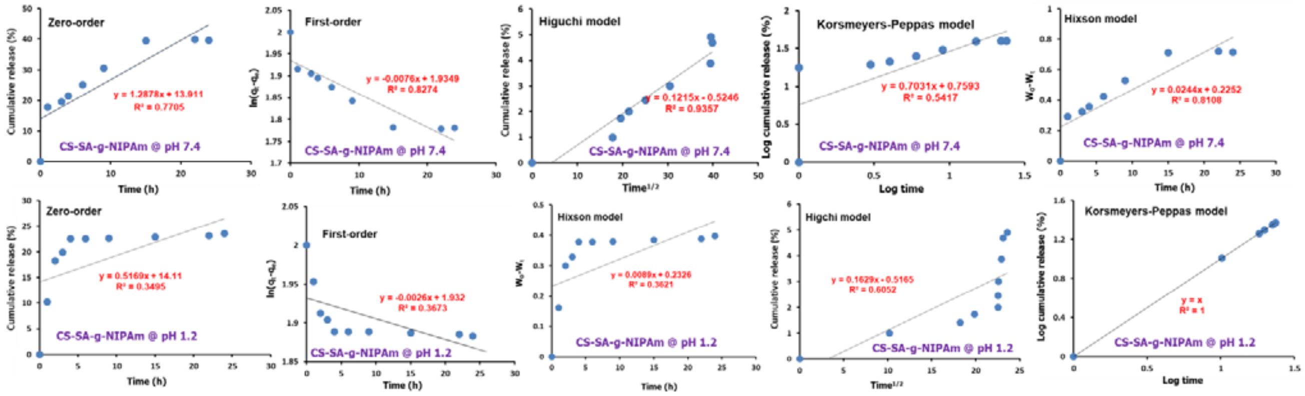

Figure S2: Kinetic studies of CS-SA-g-NIPAm.

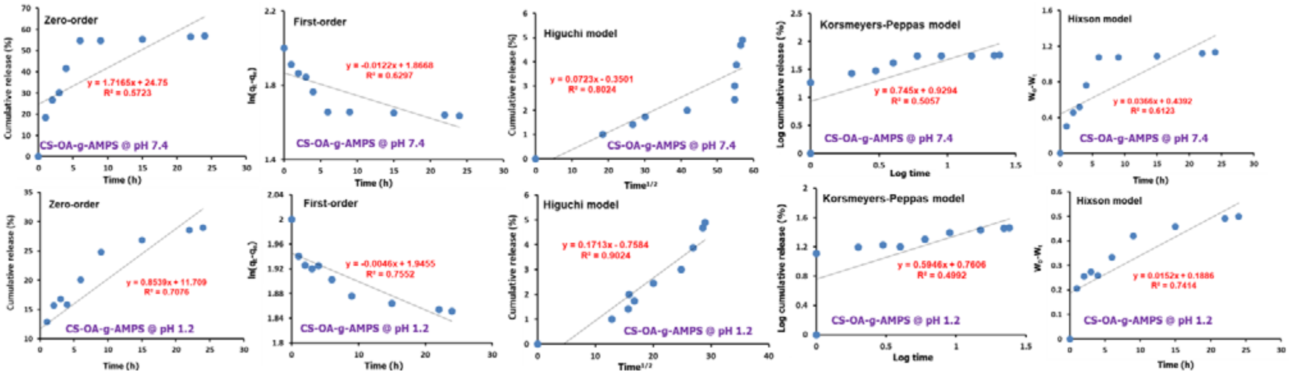

Figure S3: Kinetic studies of CS-OA-g-AMPS

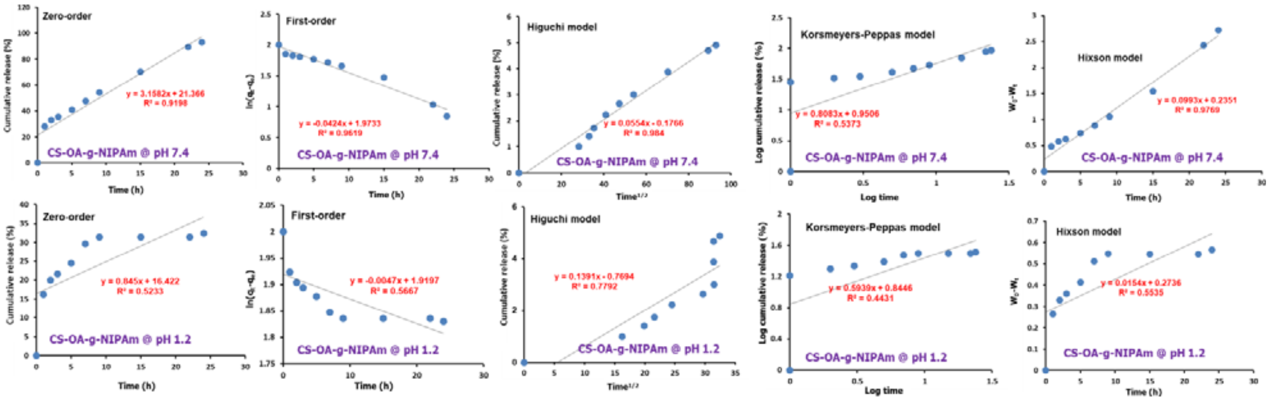

Figure S4: Kinetic studies of CS-OA-g-NIPAm.

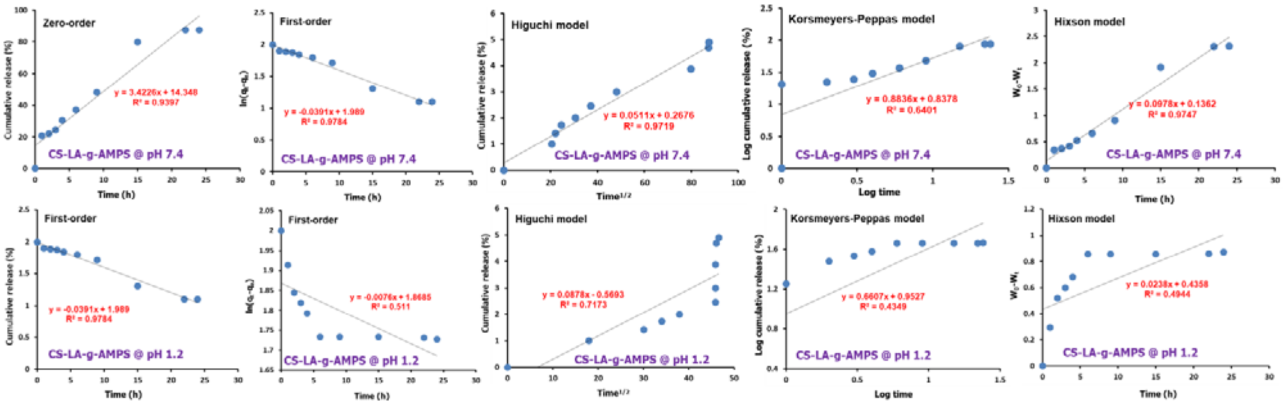

Figure S5: Kinetic studies of CS-LA-g-AMPS

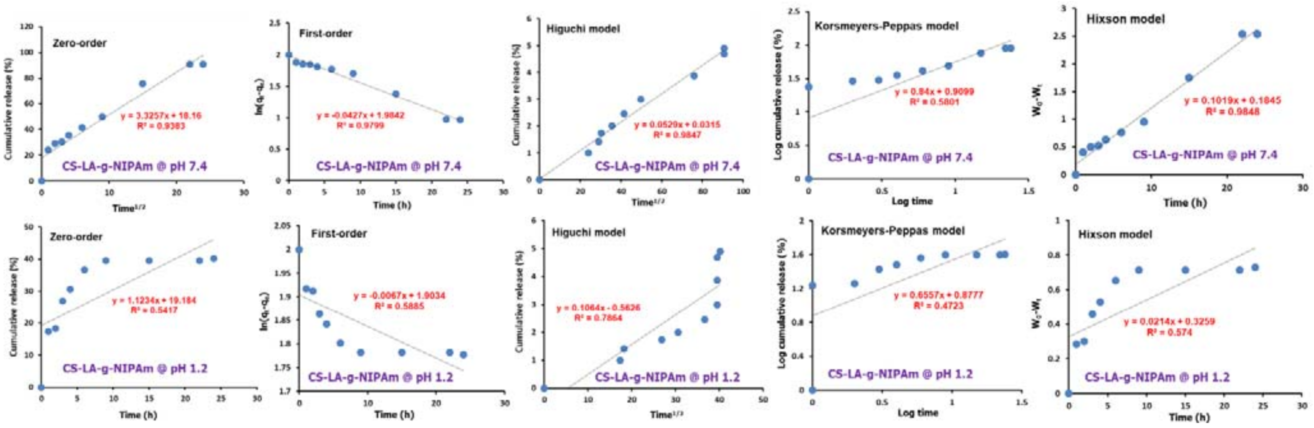

Figure S6: Kinetic studies of CS-LA-g-NIPAm.
